# HEK293T cell lines defective for O-linked glycosylation

**DOI:** 10.1371/journal.pone.0179949

**Published:** 2017-06-27

**Authors:** James M. Termini, Zachary A. Silver, Bryony Connor, Aristotelis Antonopoulos, Stuart M. Haslam, Anne Dell, Ronald C. Desrosiers

**Affiliations:** 1 Department of Pathology, University of Miami Miller School of Medicine, Miami, Florida, United States of America; 2 Department of Life Sciences, Imperial College London, London, United Kingdom; University of Liverpool, UNITED KINGDOM

## Abstract

Here we describe derivatives of the HEK293T cell line that are defective in their ability to generate mucin-type O-linked glycosylation. Using CRISPR/Cas9 and a single-cell GFP-sorting procedure, the UDP-galactose-4-epimerase (GALE), galactokinase 1 (GALK1), and galactokinase 2 (GALK2) genes were knocked out individually and in combinations with greater than 90% of recovered clones having the desired mutations. Although HEK293T cells are tetraploid, we found this approach to be an efficient method to target and disrupt all 4 copies of the target gene. Deficient glycosylation in the GALE knockout cell line could be rescued by the addition of galactose and N-acetylgalactosamine (GalNAc) to the cell culture media. However, when key enzymes of the galactose/GalNAc salvage pathways were disrupted in tandem (GALE+GALK1 or GALE+GALK2), O-glycosylation was eliminated and could not be rescued by the addition of either galactose plus GalNAc or UDP-galactose plus UDP-GalNAc. GALK1 and GALK2 are key enzymes of the galactose/GalNAc salvage pathways. Mass spectrometry was performed on whole cell lysate of the knockout cell lines to verify the glycosylation phenotype. As expected, the GALE knockout was almost completely devoid of all O-glycosylation, with minimal glycosylation as a result of functional salvage pathways. However, the GALE+GALK1 and GALE+GALK2 knockout lines were devoid of all O-glycans. Mass spectrometry analysis revealed that the disruption of GALE, GALK1, and GALE+GALK2 had little effect on the N-glycome. But when GALE was knocked out in tandem with GALK1, N-glycans were exclusively of the high mannose type. Due to the well-characterized nature of these five knockout cell lines, they will likely prove useful for a wide variety of applications.

## 1. Introduction

Cell lines that are deficient in their ability to form particular protein-linked carbohydrate have been a critical tool for the study of N- and O-linked glycans across many fields of research [1–5]. Such cell lines can provide functional insight into what effects a drastically different arrangement of glycans might have on processes such as protein folding, enzyme activity, and receptor signaling. Cell lines can be generated that alter one particular glycan linkage or result in more drastic elimination of possible glycoforms. Many of these lines have been generated by selection using toxic plant lectins or toxin-conjugated lectins specific for a particular type of glycosylation [1, 3, 6]. Cells that were able to survive the selection process now produce truncated glycans, allowing them to avoid lectin binding. Another commonly used method has involved radioactive suicide. By incorporating radioactive sugars, sulfates, and other glycan precursors into cellular glycoproteins, cell lines incapable of a particular type of glycosylation may be selected [1, 6]. These methods were often time consuming and involved large amounts of screening and characterization to identify the desired mutation. For most of these cell lines, the exact mutations that cause a particular phenotype remain unknown [1], as does the potential existence of off-site mutations. To date, most of the existing glycosylation mutant lines have been derived from the Chinese hamster ovary (CHO) cell line [1, 3–5], making them unsuitable for certain fields of research such as the study of HIV whose expression is blocked at several stages in murine cells [7, 8]. To further complicate matters, CHO cells express only a subset of the known glycosyltranserases [4, 5, 9]. This may lead to distinct differences in the glycan profile of protein produced in CHO versus human cell lines [2]. Therefore, results obtained with CHO cell lines may not accurately represent results obtained from other species-specific cell lines [9]. With the discovery of CRISPR/Cas9, the generation of extremely targeted mutations in the genes that code for key enzymes of glycosylation is now possible in any cell line with significant ease compared to traditional methods [10–12]. In less than two months, a specific mutant cell line can be generated lacking a key enzyme or glycosyltransferase as opposed to generating random undefined genetic mutations until a desired phenotype is achieved. Although there is still the possibility for off-target mutations with CRISPR/Cas9 [13, 14], due to the specificity of the gRNA, the desired phenotype is always associated with disruption of the target gene.

**Fig 1:**
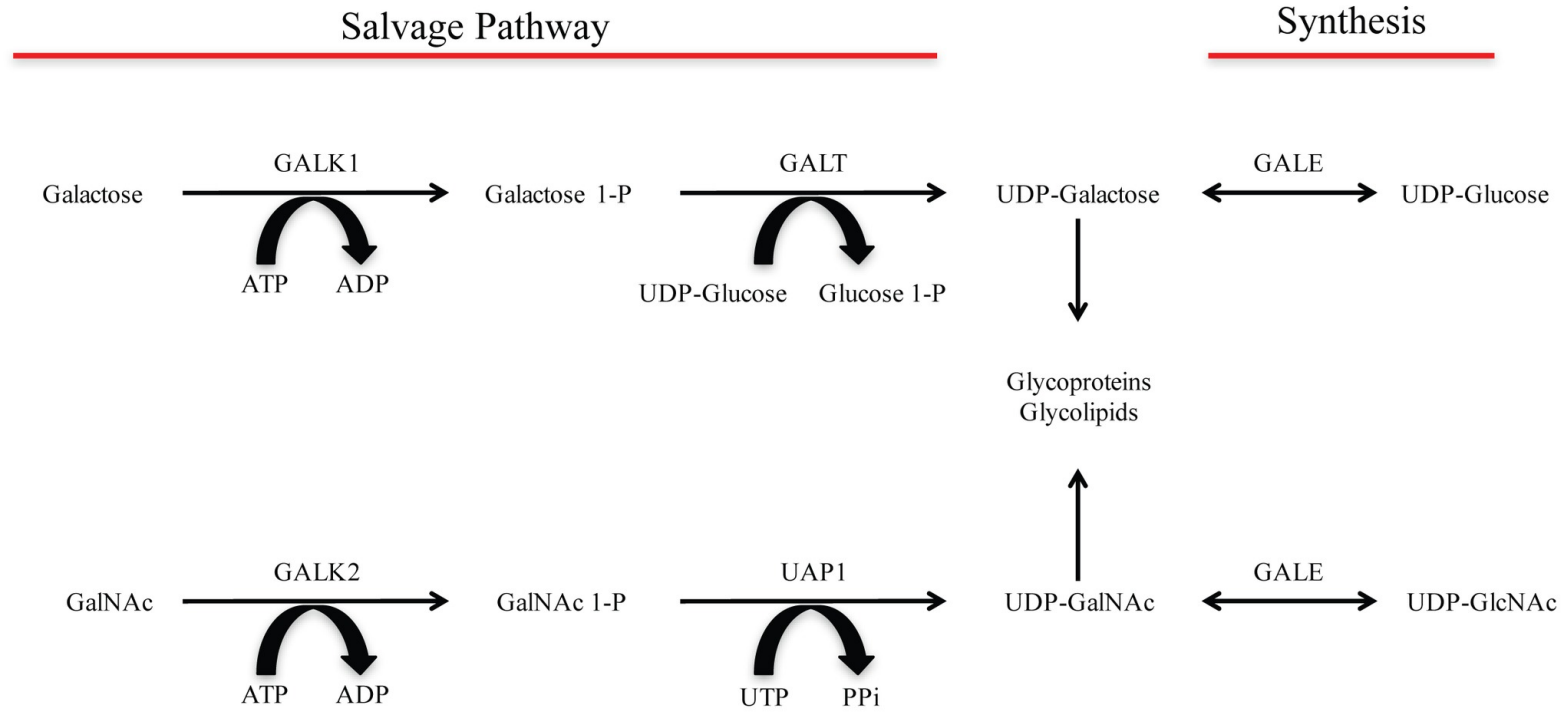
Leloir pathway of galactose metabolism. Illustrated are the 2 different pathways in which galactose and GalNAc can be salvaged or synthesized for use in glycosylation. Galactose and GalNAc can be taken up and converted to UDP-galactose and UDP-GalNAc, respectively, via the salvage pathway. UDP-galactose and UDP-GalNAc can also be interconverted from UDP-glucose and UDP-GlcNAc respectively by the enzyme UDP-galactose-4-epimerase (GALE). UDP-galactose and UDP-GalNAc can then be used for glycosylation.

The Leloir pathway of galactose metabolism is highly conserved from bacteria to humans [15]. The enzymes of the Leloir pathway are critical to generate the UDP-sugar precursors necessary for N- and O-linked glycosylation [16]. In the Leloir pathway, UDP-glucose and UDP-N-acetylglucosamine (UDP-GlcNAc) are reversibly interconverted into UDP-galactose and UDP-N-acetylgalactosamine (UDP-GalNAc) respectively, by the UDP-galactose-4-epimerase (GALE) (Fig 1) [16, 17]. These interconversions are a critical source of UDP-GalNAc and UDP-galactose for O-glycosylation. Deficiency in GALE leads to the human disorder known as galactosemia III which is associated with impaired growth, cognitive deficiencies, and even liver and renal failure [18–20]. However, both UDP-galactose and UDP-GalNAc are available via salvage pathways. Salvaged galactose can be converted to galactose 1-phosphate (galactose 1-P) by galactokinase-1 (GALK1), while salvaged GalNAc is converted to GalNAc 1-phosphate (GalNAc 1-P) by galactokinase-2 (GALK2) (Fig 1). The final step in the salvage pathways includes the conversion of galactose 1-P and GalNAc 1-P to their respective UDP forms. This is accomplished by galactose 1-phosphate uridyltransferase (GALT) and UDP-GalNAc pyrophosphorylase (UAP1) (Fig 1). Deficiencies in GALT lead to a condition known as classic galactosemia (galactosemia I). This disorder is associated with early cataract formation, mental retardation and liver dysfunction [19]. The addition of GalNAc to a serine or threonine residue via UDP-GalNAc is the necessary first step in the formation of mucin-type O-glycans, by far the major form of O-linked carbohydrate in mammalian cells.

Previously, using non-specific methods, a derivative of the Chinese hamster Ovary (CHO) cell line was developed that is devoid of GALE activity [21–23]. This GALE-deficient CHO cell line is referred to as the ldlD cell line. These cells were found to be viable in glucose-containing cell culture media despite the lack of GALE. Because these cells have a defective GALE, they cannot synthesize UDP-galactose and UDP-GalNAc. They are reliant on exogenously supplied sugars to properly glycosylate proteins. Although the ldlD cell line has been a valuable research tool, there are limitations to its use. First, since the background is hamster, ldlD cells are not suitable for certain types of research that require the use of human cells. This would include study of some human viruses that are dependent on a human cell substrate for production, entry and/or replication. In addition, due to a functional salvage pathway, there is a possibility of salvaging galactose and GalNAc from either the culture media or other glycoproteins that may be present in the media or in fetal bovine serum (FBS) [23]. Due to the high concentration of galactose in FBS as well as some commercially available media, unintended salvage may complicate data obtained from this cell line. Special care needs to be taken when selecting media conditions for experiments involving the ldlD cell line [23].

In this study, we set out to develop a set of human cell lines for the study of O-linked glycosylation. By using CRISPR/Cas9 to make targeted mutations in *GALE*, *GALK1*, and *GALK2*, we developed glycosylation-defective mammalian cell lines in a HEK293T background. These cell lines are not only phenotypically defined but also genetically defined. These cell lines are now made available to the scientific community for a broad range of possible projects.

## 2. Materials and methods

### 2.1 gRNA generation

Guide RNAs (gRNAs) (SantaCruz Biotechnology) were generated to our genes of interest: *GALE* (Gene ID: 2582), *GALK1* (Gene ID: 2584), and *GALK2* (Gene ID: 2585). Target sequences were determined using the GeCKO v2 human library. Three gRNAs per target gene were used, targeting both strands of DNA, to ensure full knockout (KO) of the gene of interest. gRNAs were cloned into an expression vector with a GFP tag to allow for single cell GFP sort.

**GALE CRISPR/Cas9 KO gRNAs**
1. CCGGGATTACATCCATGTCG
2. TCAGCTCCTGGACCCGCCGC
3. CAGGCTGGGGTTGGCGTAAC

**GALK1 CRISPR/Cas9 KO gRNAs**
1. CACAATAGCTGCCCGCGCCC
2. ACGCGCTGCTCATTGACTGC
3. TCGGTGGGCCAACTATGTCA

**GALK2 CRISPR/Cas9 KO gRNAs**
1. GAAAACGTACGCTCTCCAAC
2. GCCAAGAGTGAGCGTTACAT
3. GCCAATGTAACGCTCACTCT

### 2.2 Cell line generation

HEK293T cells (ATCC) were transfected using jetPrime transfection reagent (Polyplus-Transfection) with three gRNAs for the particular target gene. Cells were examined by GFP fluorescent microscopy at 24 hours post-transfection using a Zeiss Axio Observer A1 Microscope to gauge sufficient levels of expression necessary for downstream flow cytometric analysis and cell sorting. Axiovision software was used for image acquisition. Following microscopy, cells were harvested, washed in PBS, and resuspended in DMEM with 1mM EDTA to prevent the formation of cell aggregates. Cells were sorted on a 5 laser 17-color BD FACS SORP Aria-IIu with an Automatic Cell Deposition Unit (ACDU). The top 10% GFP-expressing cells were individually sorted into a 96-well plate. FSC-W by FSC-A and SSC-W by SSC-A were used to reduce the rate of duplets. Four hours post-sort, cells were inspected to ensure that all wells contained only one cell. Any wells that contained duplets were excluded from further processing. Once clones reached confluency in a 6-well plate, cells were lysed in RIPA buffer (Life Technologies) and used for western blot analysis.

Upon acceptance of this manuscript, GALE, GALE+GALK1, GALK1, GALE+GALK2, and GALK2 knockout cell lines will be submitted to the ATCC for use as research reagents.

### 2.3 Western blots to confirm CRISPR knockout

Cell lysate was harvested by incubating cells in RIPA buffer. Clarified lysate was loaded onto a 4–12% SDS-PAGE gel (Life Technologies). Wild-type HEK293T cell lysate was loaded in the first lane as a control. Protein was transferred to a PVDF membrane using the iBolt Dry Blotting System (Life Technologies). Membranes were probed with anti-GALE (C-4), anti-GALK1 or anti-GALK2 antibodies followed by a secondary HRP-conjugated antibody staining using the iBind western system (Life Technologies). Membranes were developed using the SuperSignal Pico Substrate (ThermoFisher) and images captured on a ImageQuant LAS 4000 mini Luminescent Image Analyzer (GE Healthcare).

### 2.4 Lectin western blots (GALE)

GALE enzymatic activity was analyzed on the protein level. GALE knockout cells (GALE KO) were transfected with an expression vector for SIVmac239 gp120 made as a truncated secreted product with a C-terminal polyhistidine tag. Secreted gp120 was purified from supernatant 48 hours post transfection using HisPur nickel-NTA columns (Thermo Fisher). 3μg of purified gp120 was run on three 4–12% SDS-PAGE gels in triplicate. The first was analyzed by Coomassie staining using the eStain^®^ 2.0 Protein Staining Device (Genscript). The second gel was probed for gp120 using 500ng/ml rhesus anti-gp120 monoclonal antibody 3.11H (purified in-house). The third gel was probed for O-glycosylation using 4μg/ml HRP-labeled Jacalin lectin (BioWorld). For protein production, cells were grown in 10% FBS, 3% lipo-depleted fetal bovine serum (LDFBS), or 3% LDFBS with galactose and GalNAc (+sugars) for 4 days until 12 hours post-transfection, at which point cells were washed and changed to serum free media. In a separate experiment, galactose and GalNAc were added to the cell culture media separately to identify whether galactose or GalNAc was the limiting precursor for O-glycosylation.

### 2.5 Lectin western blots (GALK1 and GALE+GALK1)

GALK1 enzymatic activity was analyzed on the protein level. HEK293T, GALK1 KO cells, and GALE+GALK1 double KO cells were transfected with expression vectors for SIVmac239 gp120 made as a truncated secreted product with a C-terminal polyhistidine tag. Secreted gp120 was purified from supernatant 48 hours post transfection using nickel-NTA columns. 3μg of purified gp120 was run on two 4–12% SDS-PAGE gels in duplicate. The first gel was probed for gp120 using 500ng/ml rhesus anti-gp120 monoclonal antibody 3.11H. The second gel was probed for O-glycosylation using an HRP labeled Jacalin lectin. For protein production, cells were grown in serum free media, serum free media +galactose, or serum free media +galactose +GalNAc as indicated.

### 2.6 Lectin western blots (GALK2 and GALE+GALK2)

GALK2 enzymatic activity was analyzed at the protein level. HEK293T, GALK2 KO cells, and GALE+GALK2 double KO cells were transfected with expression vectors for SIVmac239 gp120 made as a truncated secreted product with a C-terminal polyhistidine tag. Secreted gp120 was purified from supernatant 48 hours post transfection using nickel-NTA columns. 3μg of purified gp120 was run on three 4–12% SDS-PAGE gels in triplicate. The first was analyzed by Coomassie staining. The second gel was probed for gp120 using 500ng/ml rhesus anti-gp120 monoclonal antibody 3.11H. The third gel was probed for O-glycosylation using an HRP-labeled Jacalin lectin. For protein production, cells were grown in serum free media, serum free media +galactose +GalNAc (+sugars), or serum free media +UDP-galactose +UDP-GalNAc (+UDP) as indicated.

### 2.7 Sequencing

RNA was purified from mutant cell lines using the Quick-RNA Miniprep Plus Kit (Zymo Research). First-strand complementary DNA (cDNA) was synthesized by priming with oligo-DT and reverse transcribed using the Super Script IV Reverse Transcriptase (Thermo Fisher) according to manufacturer’s specified protocol.

Genes of interested were PCR amplified from cDNA using gene specific primers designed to flank areas just outside target gRNA sites and the Phusion Hot Start II High-Fidelity PCR Master Mix (Thermo Fisher) according to manufacturer’s specified protocol. Bands of the correct length were gel purified using the Zymo Gel DNA Recovery Kit (Zymo Research) and blunt-end ligated into a sequencing vector using the Zero Blunt TOPO PCR Cloning Kit (Thermo Fisher). Ligation was transformed into One Shot TOP10 Chemically Competent *E*. *coli* (Thermo Fisher) by heat shock transformation techniques. 15 colonies were grown up for each gene of interest and purified using the ZR Plasmid Miniprep kit (Zymo Research). Sequencing was performed by Genewiz on miniprep DNA using the T7 and M13R sequencing primer sites of the pCR-Blunt sequencing vector.

Upon acceptance of this manuscript, sequencing results from the GALE, GALE+GALK1, and GALE+GALK2 cell lines will be submitted to the Genbank sequence database.

### 2.8 Mass spectrometry

Glycoprotein derived N-linked and O-linked glycans were extracted from the various HEK293T cells as previously described [24]. Glycans were permethylated prior to Matrix-assisted laser desorption ionization (MALDI) MS and tandem MS (MS-MS), data were acquired using a 4800 MALDI-TOF/TOF mass spectrometer (Applied Biosystems) in the positive ion mode. Data were annotated using the glycobioinformatics tool, GlycoWorkBench [25]. The proposed assignments for the selected peaks were based on 12C isotopic composition together with knowledge of the biosynthetic pathways. The proposed structures were further confirmed using MS/MS.

### 2.9 Growth curves

HEK293T, GALE KO, GALK1 KO, GALE+GALK1 KO, GALK2 KO, and GALE+GALK2 KO cells were plated in 6 well plates on day 0. 4x10^4^ cells were used to seed wells in triplicate. Every 24 hours cells were trypsinized and counted using a Beckman Coulter Z1 Coulter Particle Counter. Measurements were taken for 4 days at which time cells reached relative confluence.

## 3. Results

### 3.1 Generation of glycosylation deficient cell lines

**Fig 2:**
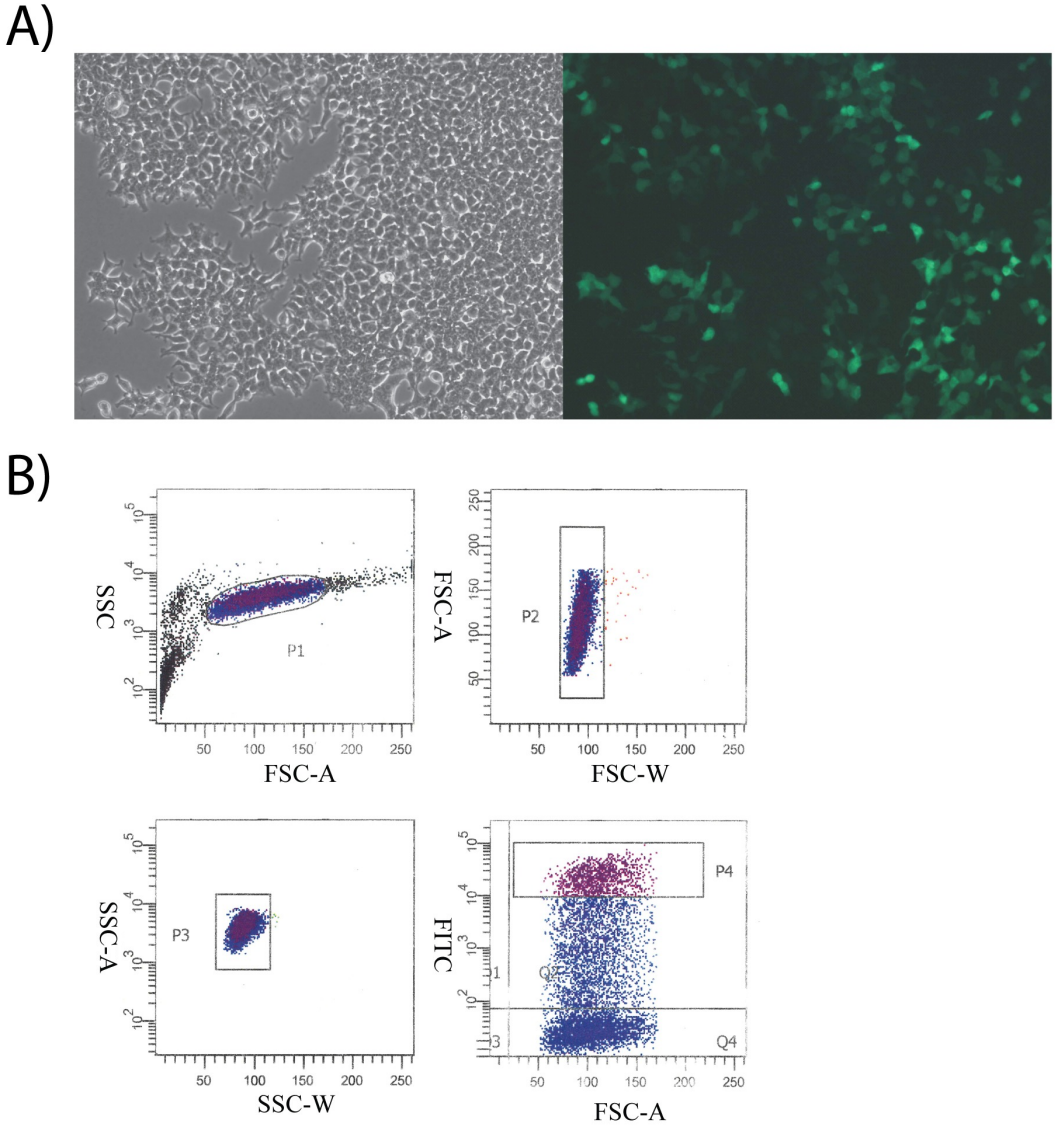
Generation of knockout cell lines using CRISPR/Cas9. Three gRNAs per target gene were cloned into an expression vector expressing both Cas9 as well as a GFP tag. **A)** Following transient transfection of HEK293T cells, GFP expression was analyzed by fluorescent microscopy at 24 hours post-transfection using a Zeiss Axio Observer A1 Microscope (20x) to determine the quality of the transfection. **B)** Gating strategy for single cell GFP sorts. The 10% highest GFP-expressing cells were sorted into 96-well plates, where one cell was sorted per well. FSC-W by FSC-A and SSC-W by SSC-A were used to reduce the rate of duplets.

DNA sequences encoding Guide RNAs (gRNAs) that target human *GALE*, *GALK1*, *and GALK2* genes were cloned into an expression vector containing a green fluorescent protein (GFP) tag. HEK293T cells were transiently transfected with three gRNA expression vectors all targeting the same gene. Cells were examined by GFP fluorescent microscopy at 24 hours post-transfection to gauge sufficient levels of expression necessary for downstream flow cytometric analysis and cell sorting (Fig 2A). Cells were harvested and sorted using a FACS Aria II cell sorter with a 96-well plate adapter. Tight gating on forward scatter width (FSC-W) by forward scatter area (FSC-A) and side scatter width (SSC-W) by side scatter area (SSC-A) was used to reduce the frequency of duplets (Fig 2B). Cells within the top 10% of GFP-expression were individually sorted, depositing an individual cell per well of a 96 well plate. Five hours post sort, wells were examined via confocal microscopy to ensure no well received more than 1 cell. Cells were allowed to grow to confluence before confirming that all copies of the target gene were disrupted.

### 3.2 Validation of GALE KO cell lines

**Fig 3:**
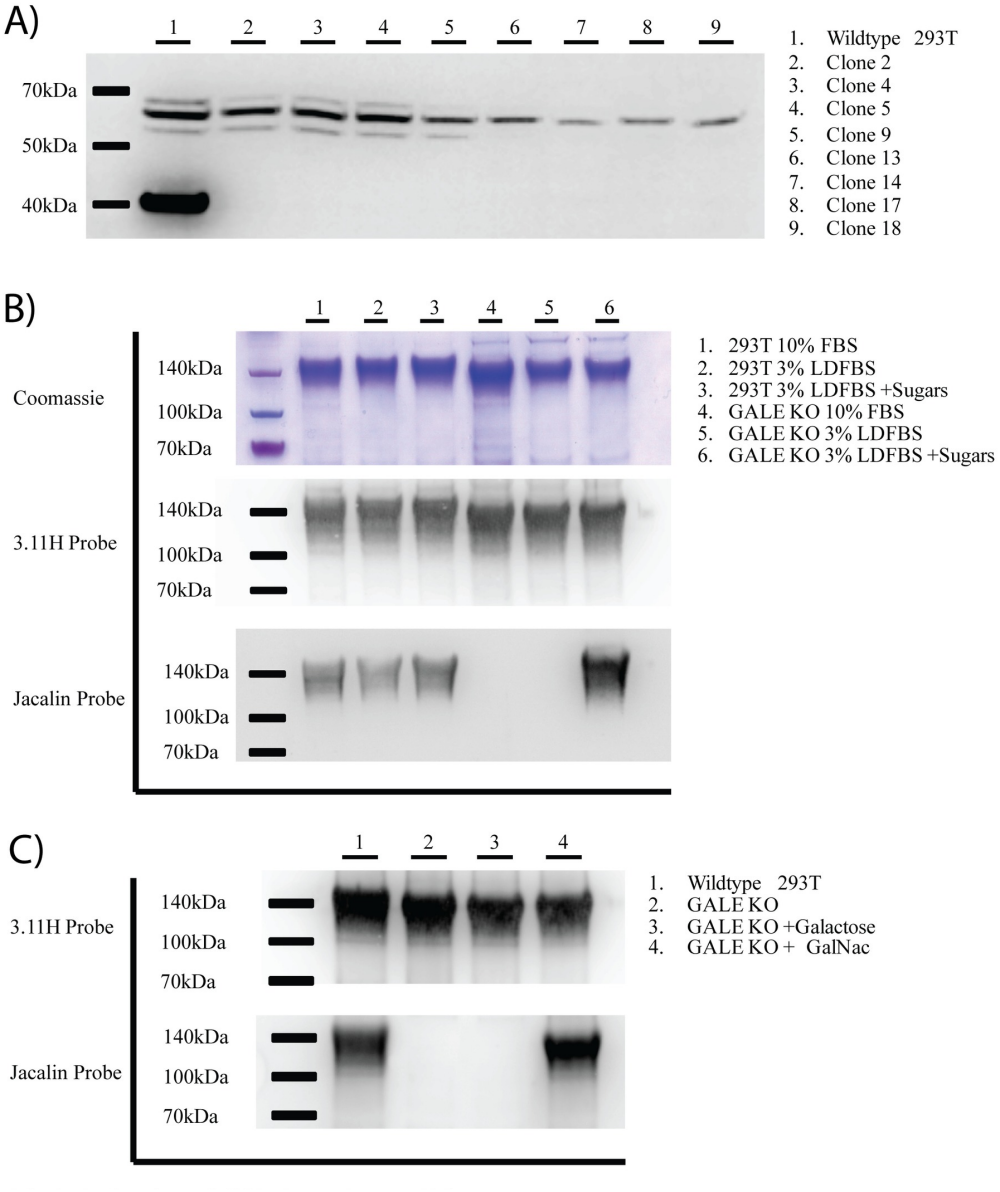
Validation of GALE knockout cell lines. Following single cell sort, potential GALE KO clones were allowed to grow until they reached confluence in 6 well plates. **A)** Cell lysate was harvested by incubating cells in RIPA buffer. Clarified lysate was loaded onto a 4–12% SDS-PAGE gel and probed with an anti-GALE antibody. Wild-type HEK293T cell lysate was loaded in the first lane as a control. **B)** GALE enzymatic activity was analyzed on the protein level. GALE KO cells (clone 4) were transfected with expression vectors for SIVmac239 gp120 made as a truncated secreted product with a C-terminal polyhistidine tag. Secreted gp120 was purified from supernatant 48 hours post transfection using nickel-NTA columns. 3μg of purified gp120 was run on three 4–12% SDS-PAGE gels in triplicate. The first was analyzed by Coomassie Blue staining. The second gel was probed for gp120 using the rhesus anti-gp120 monoclonal antibody 3.11H. The third gel was probed for O-glycosylation using an HRP-labeled Jacalin lectin. For protein production, cells were grown in 10% FBS, 3% LDFBS, or 3% LDFBS with galactose and GalNAc (+sugars) until 12 hours post-transfection, at which point cells were washed and changed to serum free media. For further detail on cell culture conditions, refer to the materials and methods. **C)** Western blots were repeated as in **B**, with one exception. Galactose and GalNAc were added to the cell culture media separately to identify whether galactose or GalNAc was the limiting precursor for O-glycosylation in our cell culture conditions.

Cell lysates from 8 HEK293T-GALE knockout clones were analyzed for the presence of full-length GALE protein by western blot. GALE protein is readily detectable by western blot at a size of 38 kDa in wildtype HEK293T cells. When cell lysates from our GALE knockout cell lines (GALE KO) were analyzed by western blot, GALE was undetectable for all 8 clones (Fig 3A). GALE KO clone 4 was sequenced for mutations in the *GALE* gene. Of the 15 cDNA clones used for sequencing, none had an intact copy of the *GALE* gene, supporting the claim that all copies of the *GALE* gene were successfully knocked out in GALE KO clone 4 (S1A and S1B Fig).

GALE KO clone 4 was chosen for further validation at the enzymatic level. To analyze if GALE KO clone 4 was indeed defective for GALE enzymatic activity, wildtype HEK293T and GALE KO cells were transiently transfected with an expression vector encoding truncated secreted SIVmac239 gp120 with a polyhistidine tag. SIVmac239 gp120 is known to contain 4 sites of O-glycosylation and to react strongly to Jacalin lectin, [26, 27] making it an ideal protein to analyze the ability of our knockout cell lines to glycosylate proteins.

GALE enzymatic activity is only one way that UDP-GalNAc and UDP-galactose is generated inside a cell. Cells can also salvage exogenous GalNAc and galactose from the cell culture media and convert these salvaged sugars into usable UDP-sugar precursors for glycosylation (Fig 1). Due to the possibility of the salvage pathway confounding our validation of the GALE KO cell line, we cultured the cells with lipo-depleted FBS (LDFBS) for 4 days prior to transfection. To rescue the phenotype, 100uM GalNAc and 10uM galactose were added to the culture media (+sugars).

3μg of purified SIV gp120 generated in parental HEK293T cells and in the GALE KO cell line were loaded onto an SDS-PAGE gel and confirmed by Coomassie Blue staining and with the rhesus anti-gp120 monoclonal antibody (3.11H) probe (Fig 3B). When probed with a Jacalin-HRP lectin to identify O-glycosylation (galactosyl (β-1,3) N-acetylgalactosamine), as expected, viral protein generated from HEK293T cells stained positive. This is due to functional GALE enzymatic activity. However, from the GALE KO clone 4, no O-glycosylated gp120 was detected in both cells grown in LDFBS as well as in normal FBS. When 100uM GalNAc and 10uM galactose were supplemented to the cell culture media, O-glycosylation was restored to the GALE KO cell line (Fig 3B lane 6), demonstrating the reversible phenotype via use of the salvage pathway.

Since FBS is known to contain high levels of galactose and glycoproteins, it was somewhat surprising that no O-glycosylation was observed in GALE KO cells grown in 10% FBS. Previously, it was found that the GALE-deficient ldlD cells can salvage sugar precursors from serum containing media to restore O-glycosylation [23]. To further examine this finding, SIVmac293 gp120 was produced in GALE KO cells cultured in the presence of 100uM GalNAc or 10uM galactose independently (Fig 3C). When cell culture media was supplemented with galactose without GalNAc, no O-glycosylation was observed on SIV gp120. However, when media was supplemented with GalNAc alone, high levels of O-glycosylation, with similar intensity to wildtype HEK293T cells, were detectable. These findings suggest that GalNAc is the limiting precursor that cannot be salvaged from the FBS-containing cell culture media. Further, due to a lack of available GalNAc and an apparent lack of salvage from glycoproteins in DMEM with 10% FBS, there appeared to be little need for the use of LD-FBS in downstream glycosylation experiments.

### 3.3 Validation of GALE+GALK1 knockout cell lines

Due to the extent of salvage we observed in the GALE KO cell lines when exposed to galactose and GalNAc, there remains the possibility that GALE KO cells grown in media conditions that unintentionally contain free galactose and GalNAc may O-glycosylate proteins. With the popularity of new proprietary media formulations, there remains a possibility of O-glycosylation confounding results. In order to simplify media conditions and erase any doubt regarding the glycoylstaion state of cell-produced proteins, we proceeded to generate cell lines with the salvage mechanisms disrupted. Salvage deficient GALE KO cell lines could prove invaluable in the study of both N- and O-linked glycosylation. Due to the large quantities of galactose in cell culture media, we decided to first develop a HEK293T-GALE+GALK1 double knockout (GALE+GALK1 KO). Both HEK293T and GALE KO cells were transfected with gRNAs for the *GALK1* gene and sorted for high GFP expression as outlined above. Since the levels of GALK1 protein were not easily detectable by western blot, potential clones were sequenced to identify clones with a disrupted *GALK1* gene (S2A and S2B Fig). To further validate these cell lines, we also examined the enzymatic activity.

**Fig 4:**
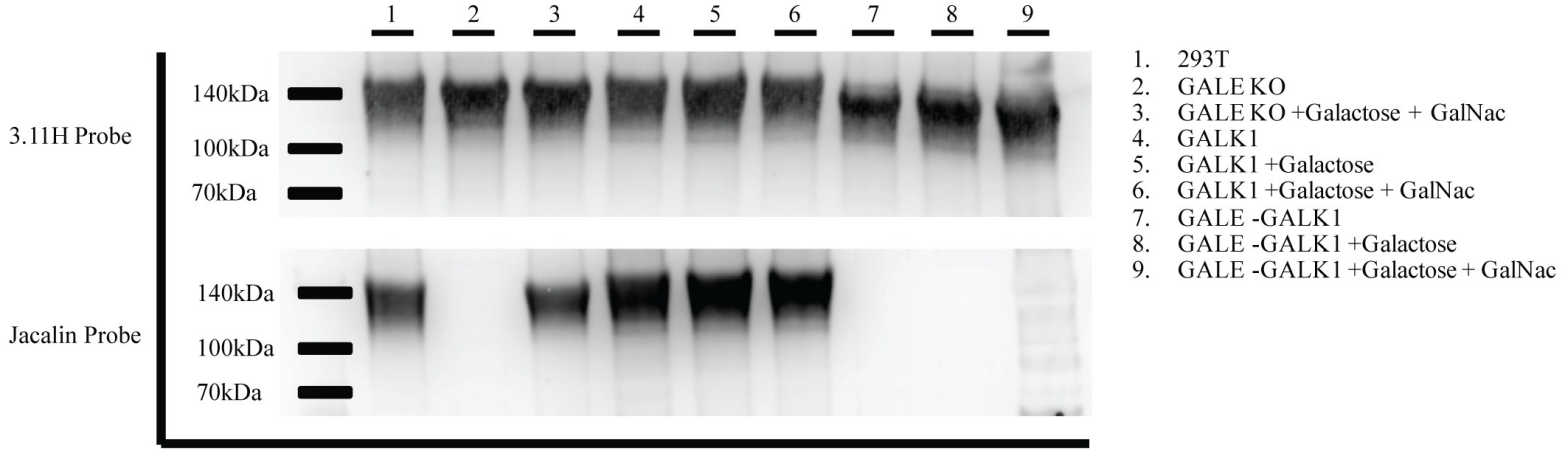
Validation of GALK1 and GALE+GALK1 deficient knockout cell lines. Following single cell sort, potential knockout clones were allowed to grow until they reached confluence in 6 well plates. GALK1 enzymatic activity was analyzed on the protein level. HEK293T, GALE KO, GALK1 KO cells (clone 10), and GALE+GALK1 double KO cells (clone 12) were transfected with expression vectors for SIVmac239 gp120 made as a truncated secreted product with a C-terminal polyhistidine tag. Secreted gp120 was purified from supernatant 48 hours post transfection using nickel-NTA columns. 3μg of purified gp120 was run on two 4–12% SDS-PAGE gels in duplicate. The first gel was probed for gp120 using the rhesus anti-gp120 monoclonal antibody 3.11H. The second gel was probed for O-glycosylation using an HRP labeled Jacalin lectin. For protein production, cells were grown in serum free media, serum free media +galactose, or serum free media +galactose +GalNAc as indicated. For further detail on cell culture conditions, refer to the materials and methods.

Truncated secreted SIV gp120 was generated in both our HEK293T-GALK1 KO and HEK293T-GALE+GALK1 KO cell lines. Since GALK1 is a critical enzyme in the salvage of galactose, we cultured each line with either galactose alone or GalNAc and galactose. As expected, GALK1 KO cells produced fully glycosylated SIV gp120 in all culture conditions (Fig 4). This is due to a functional copy of the *GALE* gene. However, when GALK1 was knocked out in combination with GALE, no O-glycosylation of SIV gp120 was observed in all culture conditions. Since no O-glycosylation was observed when GALE+GALK1 KO cells were cultured with the addition of GalNAc and galactose, we can conclude that all copies of the *GALK1* gene were appropriately disrupted.

### 3.4 Validation of GALE+GALK2 knockout cell lines

The addition of GalNAc to a threonine or serine is the first step in all forms of mucin-type O-glycosylation. Since it remains possible that the GALE KO cell line could salvage a small amount of GalNAc from glycoproteins present in FBS, we decided that a HEK293T-GALE+GALK2 double knockout cell line (GALE+GALK2 KO) would be a useful tool for experiments when it is imperative to use a cell line devoid of all O-glycosylation. With this cell line, there would be no concern of salvage of GalNAc in all media conditions.

**Fig 5:**
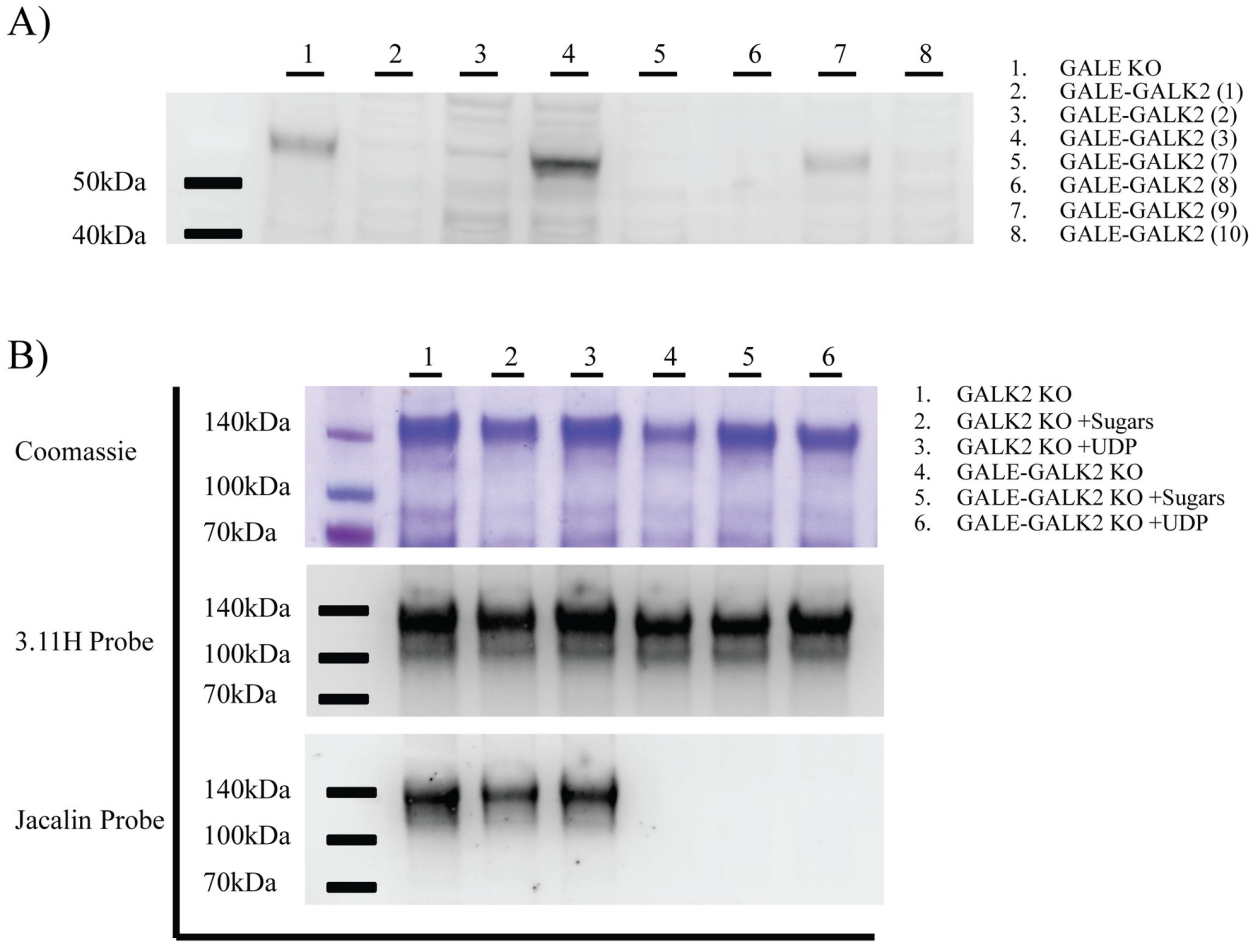
Validation of GALK2 and GALE+GALK2 deficient knockout cell lines. Following single cell sort, potential knockout clones were allowed to grow until they reached confluence in 6 well plates. **A)** Cell lysate was harvested by incubating cells in RIPA buffer. Clarified lysate was loaded onto a 4–12% SDS-PAGE gel and probed with an anti-GALK2 antibody. Parental GALE KO cell lysate was loaded in the first lane as a control. **B)** GALK2 enzymatic activity was analyzed at the protein level. GALK2 KO cells (clone 7) and GALE+GALK2 double KO cells (clone 7) were transfected with expression vectors for SIVmac239 gp120 made as a truncated secreted product with a C-terminal polyhistidine tag. Secreted gp120 was purified from supernatant 48 hours post transfection using nickel-NTA columns. 3μg of purified gp120 was run on three 4–12% SDS-PAGE gels in triplicate. The first was analyzed by Coomassie Blue staining. The second gel was probed for gp120 using the rhesus anti-gp120 monoclonal antibody 3.11H. The third gel was probed for O-glycosylation using an HRP labeled Jacalin lectin. For protein production, cells were grown in serum free media, serum free media +galactose +GalNAc (+Sugars), or serum free media +UDP-galactose +UDP-GalNAc (+UDP) as indicated. For further detail on cell culture conditions, refer to the materials and methods.

Following transfection, GFP sort, and expansion, cell lysate from 7 GALE+GALK2 knockout clones was analyzed for the presence of full-length GALK2 protein by western blot. GALK2 protein was detectable by western blot at a size of 61kDa in wildtype HEK293T cells. When cell lysate from our GALE+GALK2 knockout cell lines was analyzed by western blot, no presence of GALK2 was detectable in lanes 2, 3, 5, 6, and 8 (Fig 5A). GALE+GALK2 clone 7 (lane 5) was chosen for further analysis.

Clones for both HEK293T-GALK2 (clone 7) and HEK293T-GALE+GALK2 (clone 7) were sequenced for mutations in the *GALK2* gene. Of the 15 cDNA clones sent for each cell line, none had an intact copy of the *GALK2* gene, further supporting the claim that the GALK2 was successfully knocked out (S3A and S3B Fig).

Monomeric secreted SIV gp120 was generated in both the GALK2 (clone 7) and GALE+GALK2 (clone 7) knockout cell lines. Since GALK2 is a critical enzyme in the salvage of GalNAc, we cultured each line with galactose and GalNAc (Fig 5B lanes 2&5) to test the cell lines’ ability to salvage. As expected, GALK2 KO cells fully glycosylated SIV gp120 in all culture conditions (Fig 4). This is due to a functional copy of the *GALE* gene. However, when GALK2 was knocked out in combination with GALE, no O-glycosylation was observed in all culture conditions. Since no O-glycosylation was observed with GALE+GALK2 KO clone 7 cultured with the addition of GalNAc and galactose, we can conclude that all copies of the *GALK2* gene were correctly knocked out.

GALK2 and GALE+GALK2 KO cell lines were also cultured in the presence of 100uM UDP-GalNAc and 10uM UDP-galactose. Since both GalNAc and galactose must be converted into their respective UDP-precursors before being utilized for glycosylation, we were interested in whether the UDP forms of the sugars could be salvaged and the knockout phenotype could thus be rescued. However, even at high concentrations of UDP-sugar precursors, no restoration of O-glycosylation was observed by lectin western blot (Fig 5B Lane 6). Although UDP-galactose and UDP-GalNAc transporters are known to transport sugars from the cytoplasm to the golgi and endoplasmic reticulum, HEK-293T cells appear unable to transport these UDP-precursors across the plasma membrane. Also, endocytosed UDP-precursors do not appear to make it to the golgi intact, most likely due to degradation in intracellular vesicles.

### 3.5 Mass spectrometry analysis of knockout cell lines

**Fig 6:**
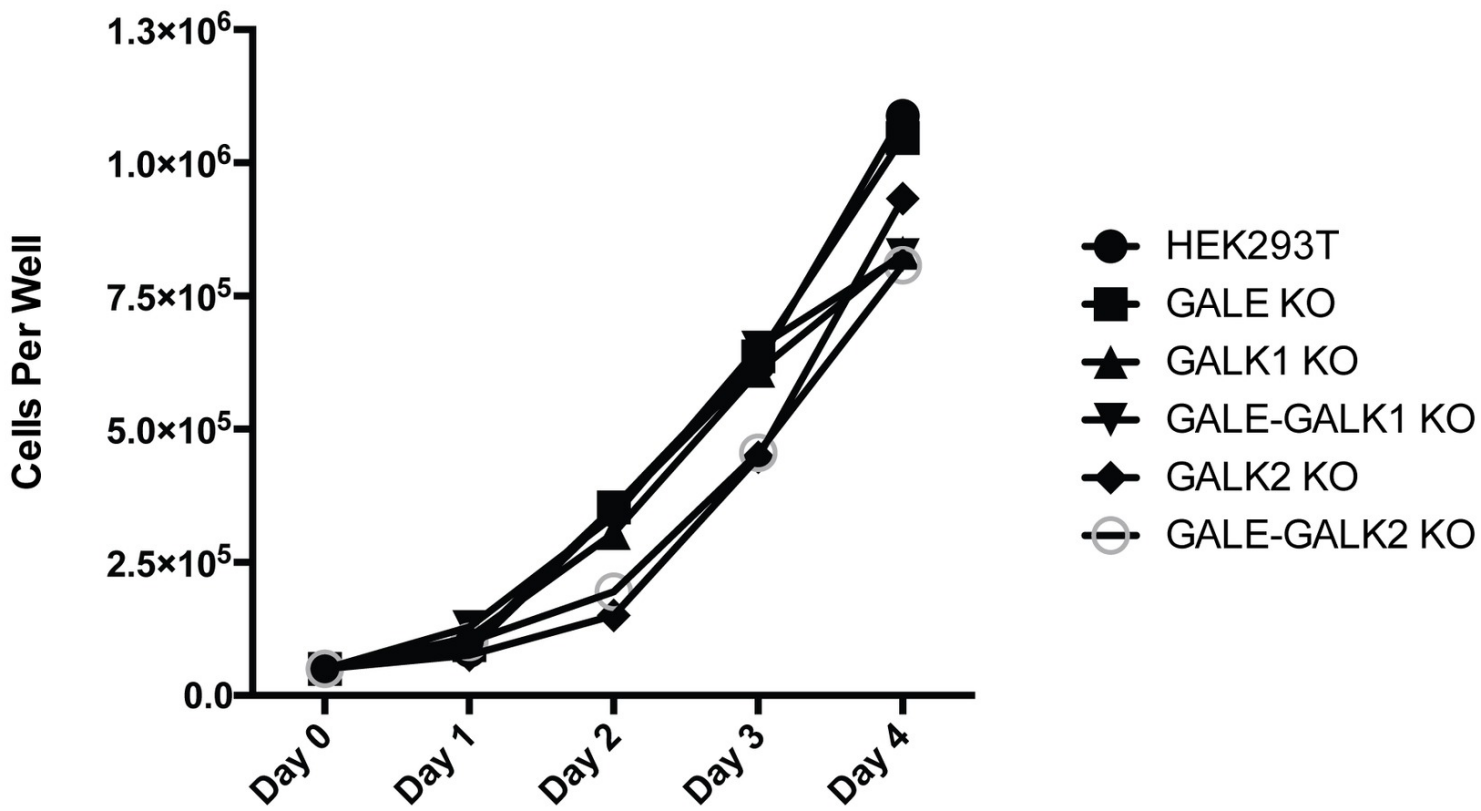
Cell growth curve. HEK293T cells, GALE KO (clone 4), GALK1 KO (clone 10), GALE+GALK1 KO (clone 12), GALK2 KO (clone 7), and GALE+GALK2 KO (clone 7) were analyzed to determine if disrupting key enzymes of the Leloir pathway impacted cell growth. 4x10^4 HEK293T cells were plated in triplicate in 6-well plates starting on day 0. Cells were harvested and counted using a Beckman Coulter Z1 Coulter Particle Counter every 24 hours.

Due to the established importance of O-glycosylation to support life and the known medical disorders associated with defects in GALE and galactokinases, it was somewhat surprising to find that our knockout cell lines devoid of all O-glycosylation grew equally well as the wildtype HEK293T cells (Fig 6). Even after long culture periods in the presence of galactose, there was no evidence to suggest a toxic build up of galactose 1-P as is observed in human conditions. To further investigate these findings, we decided to characterize the glycosylation capabilities of these cell lines. Using mass spectrometry, we analyzed the N-linked and O-linked glycans present in our cell lines. This allowed us to fully confirm that our cell lines had all copies of the target gene fully disrupted. HEK293T cells, HEK293T-GALE KO, HEK293T-GALK1 KO, HEK293T-GALE+GALK1 KO, HEK293T-GALE+GALK2 KO, and HEK293T-GALE+GALK1 KO cells +galactose were repeatedly washed with PBS prior to mass spectrometry analysis.

**Fig 7:**
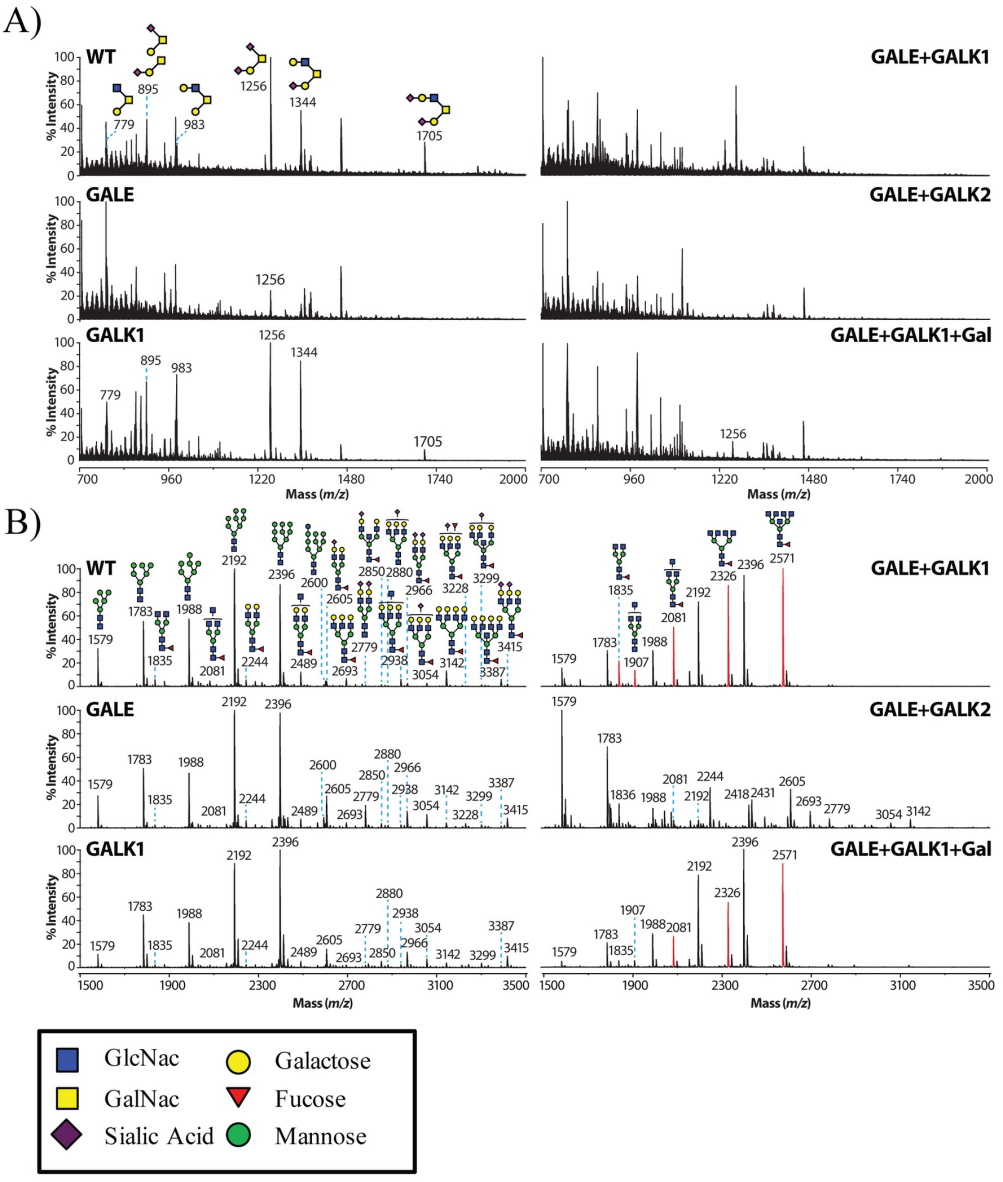
Mass spectrometry analysis of total cell lysate. HEK293T cells, GALE KO (clone 4), GALK1 KO (clone 10), GALE+GALK1 KO (clone 12), GALE+GALK2 KO (clone 7), and GALE+GALK1 KO cells +galactose were grown in standard DMEM-10% FBS media. Cells were washed 3 times in cold PBS before analysis. **A)** MALDI-TOF mass spectra of permethylated O-glycans isolated from total cell lysates. All molecular ions are [M+Na]+. The sugar symbols are those as described in [28]. Structural assignments are based on monosaccharide composition (obtained by MALDI-TOF MS), fragmentation analyses (MALDI-TOF/TOF MS/MS), and knowledge of glycan biosynthetic pathways. All peakes not labeled with am/z value are not glycans and are either matrix or general chemical background peaks. **B)** MALDI-TOF MS profiles of the permethylated N-linked glycans from total cell lysate of HEK293T cells, GALE KO, GALK1 KO, GALE+GALK1 KO, GALE+GALK2 KO, and GALE+GALK1 KO cells +galactose. All molecular ions are present in sodiated form [M +Na]^+^.

Matrix-assisted laser desorption/ionization-time-of-flight (MALDI-TOF) mass spectra of permethylated O-glycans isolated from total cell lysate were recorded. As expected, HEK293T and GALK1 KO cells displayed a normal range of O-glycans (Fig 7A). Consistent with lectin western blot of SIV gp120, both GALE+GALK1 and GALE+GALK2 KO cells had no detectable O-glycans. GALE KO was almost completely devoid of all O-glycosylation with minimal glycosylation possibly as a result of functional salvage pathways. Of note, in GALE+GALK1 +galactose, there was a very low level signal for disialyated core 1 observed (m/z 1256). However, the data suggest that this is most likely due to residual FBS contamination from the cell culture media and not a functional salvage pathway. The lack of O-glycans in the GALE+GALK1 sample as well as a lack of complex N-glycans in the GALE+GALK1 +galactose (Fig 7B) suggests a complete disruption of both target genes.

MALDI-TOF MS profiles of the permethylated N-linked glycans from total cell lysate of HEK293T cells, HEK293T-GALE KO, HEK293T-GALK1 KO, HEK293T-GALE+GALK1 KO, HEK293T-GALE+GALK2 KO, and HEK293T-GALE+GALK1 KO cells +galactose were also generated. Major peaks are annotated with relevant N-glycan structures shown in symbol form (Fig 7B). Wildtype HEK293T cells displayed both high mannose and galactose containing bi-, tri- and tetra-antennary complex N-glycans. Of interest, GALE+GALK1 KO and GALE+GALK1 KO +galactose cells appear to have high mannose N-glycans and an increased expression of bi-, tri- and tetra-antennary truncated complex N-glycans that lack galactose (m/z 1835, 1907, 2081 2326 and 2571), due to a lack of usable UDP-galactose in the cells. GALE KO cells, although being devoid of epimerase activity, still displayed galactose containing complex N-glycans (Fig 7B). As expected GALK1 had less complex N-glycans when compared to wildtype HEK293T cells. These subtle changes are more evident when the high molecular weight galactose containing complex N-glycans are viewed (S4 Fig). This is most likely due to the lack of galactose salvage in these cells. These data confirm the findings of our lectin western blots and complement the conclusion that target genes in our knockout cell lines were completely disrupted, and confirm predictions on the effects of these knockouts on glycosylation patterns.

## 4. Discussion

Although glycosylation-deficient cell lines have been used for decades, to our knowledge, this is the first report using CRISPR/Cas9 technology to make human cell lines with drastic alterations to the O-linked glycosylation machinery. Compared to traditional methods of lectin based selection or radioactive suicide, CRISPR/Cas9 proved to be a far superior method for the generation of glycosylation deficient cell lines [12]. With the exception of our GALE+GALK2 KO, all selections done by single-cell GFP-sorting for the purpose of fluorescent enrichment yielded greater than 90% positive knockout clones. The speed with which knockout cell lines can be generated makes this approach superior to lectin-based methods. Instead of a lengthy process to validate the glycosylation phenotype, clones with a genetic mutation in the gene of interest are generated and validated in a matter of months. As demonstrated by our sequencing data (S1–S3 Figs), CRISPR/Cas9 is highly efficient at disrupting target genes at sites in close proximity to the gRNA, resulting in premature stop codons in most cases.

One somewhat surprising finding from our data was that even the most extreme double knockout (GALE+GALK2), which was shown to be devoid of all O-glycosylation (Figs 5B and 7A), was viable and exhibited normal growth rates (Fig 6). Deficiency in GALE leads to the human disorder known as galactosemia III which is associated with impaired growth, cognitive deficiencies, and liver and renal failure [17–19]. Of note, cells derived from even the most adversely affected GALE-deficient patients are not completely GALE null. These patients tend to have residual GALE enzymatic activity [17, 18, 20, 29, 30]. Although it is believed that complete loss of GALE enzymatic activity is incompatible with life [18], both our GALE and GALE+GALK2 KO cell lines thrive in the complete absence of these enzymes (Fig 6).

These data indicate that mucin-type O-glycosylation may not necessarily be critical for the health and survival of an individual cell. This hypothesis is supported by previous work done on the ldlD cell line, as well as the work of Schulz *et al*. [31]. When the ldlD CHO cell line is grown in media containing lipo-depleted serum, no O-linked glycosylation can be detected. However, consistent with our findings, the cells do appear to be viable in culture (Fig 6) [21, 23]. Schulz *et al*. found that UDP-GalNAc was not required for cell proliferation [31]. The generation of the ldlD [eGALE] cell line, a GALE-deficient cell line engineered to express *E*. *coli* GALE lacking the UDP-GlcNAc -> UDP-GalNAc activity, demonstrated that the apparent growth impairment of GALE-deficient cells exposed to high levels of galactose was completely independent of the UDP-GalNAc deficiency. Schulz *et al*. therefore concluded that this impairment is likely independent of *O-*linked glycosylation defects [31].

Similar to the CHO-ldlD cell line generated by the laboratory of Monty Krieger, our GALE KO cell line has the same restorable defect in O-glycosylation [21–23]. With the addition of galactose and GalNAc to the culture media, salvage pathways are able to compensate for the lack of GALE enzymatic activity and restore O-linked glycosylation [21]. Krieger *et al*. also published that the ldlD cell line can restore O-glycosylation when cultured in 10% FBS; lipo-depleted serum was necessary to minimize unwanted O-glycosylation via the salvage pathway [23]. Unlike these findings, there appears to be little need to use lipo-depleted serum when culturing our GALE-deficient cells to achieve a glycosylation-free state. When GALE KO cells were cultured in standard 10% FBS-containing media, no O-glycosylation was observed by western blot (Fig 2B) and a very minimal peak was observed by mass spectrometry (Fig 7A). However, to make these cells easier to work with and to remove any doubt about possible salvage in standard cell culture conditions, we generated the GALE+GALK1 and GALE+GALK2 double knockout cell lines. We observed that even with the addition of galactose or GalNAc, respectively, these cell lines were unable to restore O-glycosylation (Figs 4 and 5B), which simplifies their use for standard experiments. No care is necessary to ensure that all components of cell culture media are glycoprotein-free as well as lacking galactose and GalNAc. With the rise of “proprietary formulations” for serum-free media, this can prove difficult.

Since the cell lines generated in this publication are all on the HEK293T background, they are broadly applicable to human research. These cell lines may be used directly to study the impact of O-glycosylation on the function of individual human proteins without the need for transient transfection or the generation of stable cell lines. Also, these cell lines simplify the study of viruses in an O-glycan deficient state. Many human viruses cannot be grown or packaged in a CHO cell line. With the ease of growth and transfection of the HEK293T cell line, these concerns are no longer relevant in our knockout lines. Due to the well-characterized nature of these five knockout cell lines, they will likely prove valuable for the study of glycosylation in a wide variety of fields.

## Supporting information

S1 Fig Sequencing of the GALE deficient cell line. **A)** Three unique guide RNAs (light blue, underlined nucleotides) were designed to target *GALE* and generate a deficient HEK293T cell line. To confirm the cell lines, we extracted RNA from 5x10^6^ HEK293T cell lines that had been knocked out for GALE and deemed promising by western blot. cDNA was generated using the superscript IV first-strand synthesis system and amplified a region of the *GALE* mRNA transcript that is conserved across all transcript isoforms. We performed a PCR cleanup and cloned the *GALE* transcripts using the Zero Blunt TOPO PCR Cloning Kit. Because HEK293T cells are a tetraploid cell line, we sent 15 unique colonies for Sanger sequencing to ensure that no functional transcripts were present. No functional transcripts were identified. Three unique nucleotide sequences were found in the area of gRNA #1, two were found in the area of gRNA #2, and two were found in the area of gRNA #3. **B)** Upon conversion of these *GALE* mRNA transcripts to protein sequences, premature stop codons were consistently identified prior to the 60^th^ amino acid of the GALE protein.
(TIF)

S2 Fig Sequencing of the galactokinase-1 (GALK1) deficient cell line. **A)** Three unique guide RNAs (light blue, underlined nucleotides) were designed to target *GALK1* and generate a deficient HEK293T cell line. To confirm the cell lines, we extracted RNA from 5x10^6^ HEK293T cell lines that had been knocked out for GALK1. We created cDNA using the superscript IV first-strand synthesis system and amplified a region of the *GALK1* mRNA transcript that is conserved across all transcript isoforms. We performed a PCR cleanup and cloned the *GALK1* transcripts using the Zero Blunt TOPO PCR Cloning Kit. Because HEK293T cells are a tetraploid cell line, we sent 15 unique colonies for Sanger sequencing to ensure that no functional transcripts were present in the cell line. Mutations were present in close proximity to all three gRNA sites. We observed three unique mutated transcripts in the region of gRNA #1, one unique transcript in the region of gRNA #2, and three unique transcripts in the region of gRNA #3. No wild-type *GALK1* transcripts were found in any of the clones. **B)** Translation of the mRNA sequences yielded only non-functional, truncated protein sequences. Three unique protein sequences were all identified, each one containing a premature stop codon.
(TIF)

S3 Fig Sequencing of the galactokinase-2 (GALK2) deficient cell line. **A)** Three unique guide RNAs (light blue, underlined nucleotides) were designed to target *GALK2* and generate a deficient HEK293T cell line. To confirm the cell lines, we extracted RNA from 5x10^6^ HEK293T cell lines that had been knocked out for GALK2 and deemed promising by western blot. We created cDNA using the superscript IV first-strand synthesis system and amplified a region of the *GALK2* mRNA transcript that is conserved across all transcript isoforms. We performed a PCR cleanup and cloned the *GALK2* transcripts using the Zero Blunt TOPO PCR Cloning Kit. Because HEK293T cells are a tetraploid cell line, we sent 15 unique colonies for Sanger sequencing to ensure that no functional transcripts were present. All of the clones displayed an identical set of nucleotide mutations in the area of the first gRNA, while two highly similar yet unique sets of mutations were identified circa the second gRNA. Interestingly, no mutations were found near the third gRNA. **B)** Upon conversion of the mRNA transcripts to protein sequences, premature stop codons leading to truncated proteins were observed across all clones.
(TIF)

S4 Fig Mass spectrometry analysis of N-glycans in total cell lysate. Matrix-assisted laser desorption/ionization-time-of-flight (MALDI-TOF) mass spectra profiles of high molecular weight permethylated N-glycans isolated from total cell lysate of HEK293T (upper panel), GALE KO (middle panel), GALK1 KO (lower panel) cells. All molecular ions are [M+Na]^+^. Profiles of N-glycans are from the 50% acetonitrile fraction from a C18 sep-pak. The sugar symbols are those as described in (Varki et al., 2015). Putative structural based on monosaccharide composition (obtained by MALDI-TOF MS), fragmentation analyses (MALDI-TOF/TOF MS/MS), and knowledge of glycan biosynthetic pathways. For non-annotated peaks on GALE KO and GALK1 KO see structural assignments on HEK293T.
(TIF)

## Abbreviations

Cas9: CRISPR associated protein 9
CRISPR: Clustered regularly interspaced short palindromic repeats
GALE: UDP-galactose-4-epimerase
GALK1: galactokinase 1
GALK2: galactokinase 2
GalNAc: N-acetylgalactosamine
GlcNAc: N-acetylglucosamine
GlmU: N-acetylglucosamine-1-phosphate uridyltransferase
gRNA: Guide RNA
LDFBS: Lipo-depleted fetal bovine serum
MALDI: Matrix-assisted laser desorption ionization
MALDI-TOF: Matrix-assisted laser desorption/ionization-time-of-flight

## References

[1] Stanley P. Glycosylation mutants of animal cells. Annual review of genetics. 1984;18:525–52. Epub 1984/01/01. doi: 10.1146/annurev.ge.18.120184.002521 . 6241454 10.1146/annurev.ge.18.120184.002521

[2] Butler M, Spearman M. The choice of mammalian cell host and possibilities for glycosylation engineering. Current opinion in biotechnology. 2014;30:107–12. Epub 2014/07/10. doi: 10.1016/j.copbio.2014.06.010 . 25005678 10.1016/j.copbio.2014.06.010

[3] Patnaik SK, Stanley P. Lectin-resistant CHO glycosylation mutants. Methods in enzymology. 2006;416:159–82. Epub 2006/11/23. doi: 10.1016/S0076-6879(06)16011-5 . 17113866 10.1016/S0076-6879(06)16011-5

[4] Stanley P. Glycosylation engineering. Glycobiology. 1992;2(2):99–107. Epub 1992/04/01. . 1606361 10.1093/glycob/2.2.99

[5] Stanley P, Raju TS, Bhaumik M. CHO cells provide access to novel N-glycans and developmentally regulated glycosyltransferases. Glycobiology. 1996;6(7):695–9. Epub 1996/10/01. . 8953280 10.1093/glycob/6.7.695

[6] Varki A, Cummings RD, Esko JD, Stanley P, Hart G, Aebi M, et al Essentials of Glycobiology. Cold Spring Harbor (NY): Cold Spring Harbor Laboratory Press; 2015 doi: 10.1093/glycob/cwv091 27010055

[7] Rehwinkel J. Mouse knockout models for HIV-1 restriction factors. Cellular and Molecular Life Sciences. 2014;71(19):3749–66. doi: 10.1007/s00018-014-1646-8 ; 24854580 10.1007/s00018-014-1646-8 PMC4160573

[8] Bloch N, Glasker S, Sitaram P, Hofmann H, Shepard CN, Schultz ML, et al A Highly Active Isoform of Lentivirus Restriction Factor SAMHD1 in Mouse. The Journal of biological chemistry. 2017;292(3):1068–80. Epub 2016/12/07. doi: 10.1074/jbc.M116.743740 ; 27920203 10.1074/jbc.M116.743740 PMC5247641

[9] Hossler P, Khattak SF, Li ZJ. Optimal and consistent protein glycosylation in mammalian cell culture. Glycobiology. 2009;19(9):936–49. Epub 2009/06/06. doi: 10.1093/glycob/cwp079 . 19494347 10.1093/glycob/cwp079

[10] Cong L, Ran FA, Cox D, Lin S, Barretto R, Habib N, et al Multiplex Genome Engineering Using CRISPR/Cas Systems. Science (New York, NY). 2013;339(6121):819–23. doi: 10.1126/science.1231143 ; 23287718 10.1126/science.1231143 PMC3795411

[11] Mali P, Yang L, Esvelt KM, Aach J, Guell M, DiCarlo JE, et al RNA-Guided Human Genome Engineering via Cas9. Science (New York, NY). 2013;339(6121):823–6. doi: 10.1126/science.1232033 ; 23287722 10.1126/science.1232033 PMC3712628

[12] Cherepanova NA, Gilmore R. Mammalian cells lacking either the cotranslational or posttranslocational oligosaccharyltransferase complex display substrate-dependent defects in asparagine linked glycosylation. Scientific reports. 2016;6:20946 Epub 2016/02/13. doi: 10.1038/srep20946 ; 26864433 10.1038/srep20946 PMC4750078

[13] Cho SW, Kim S, Kim Y, Kweon J, Kim HS, Bae S, et al Analysis of off-target effects of CRISPR/Cas-derived RNA-guided endonucleases and nickases. Genome Research. 2014;24(1):132–41. doi: 10.1101/gr.162339.113 ; 24253446 10.1101/gr.162339.113 PMC3875854

[14] Lin Y, Cradick TJ, Brown MT, Deshmukh H, Ranjan P, Sarode N, et al CRISPR/Cas9 systems have off-target activity with insertions or deletions between target DNA and guide RNA sequences. Nucleic Acids Research. 2014;42(11):7473–85. doi: 10.1093/nar/gku402 24838573 10.1093/nar/gku402 PMC4066799

[15] Holden HM, Rayment I, Thoden JB. Structure and function of enzymes of the Leloir pathway for galactose metabolism. The Journal of biological chemistry. 2003;278(45):43885–8. Epub 2003/08/19. doi: 10.1074/jbc.R300025200 . 12923184 10.1074/jbc.R300025200

[16] Frey PA. The Leloir pathway: a mechanistic imperative for three enzymes to change the stereochemical configuration of a single carbon in galactose. The FASEB Journal. 1996;10(4):461–70. 8647345

[17] Timson DJ. The structural and molecular biology of type III galactosemia. IUBMB life. 2006;58(2):83–9. Epub 2006/04/14. doi: 10.1080/15216540600644846 . 16611573 10.1080/15216540600644846

[18] Fridovich-Keil J, Bean L, He M, Schroer R. Epimerase Deficiency Galactosemia In: Pagon RA, Adam MP, Ardinger HH, Wallace SE, Amemiya A, Bean LJH, et al, editors. GeneReviews(R). Seattle (WA): University of Washington, Seattle University of Washington, Seattle GeneReviews is a registered trademark of the University of Washington, Seattle. All rights reserved.; 1993.

[19] Lai K, Elsas LJ, Wierenga KJ. Galactose toxicity in animals. IUBMB life. 2009;61(11):1063–74. Epub 2009/10/28. doi: 10.1002/iub.262 ; 19859980 10.1002/iub.262 PMC2788023

[20] Openo KK, Schulz JM, Vargas CA, Orton CS, Epstein MP, Schnur RE, et al Epimerase-Deficiency Galactosemia Is Not a Binary Condition. American journal of human genetics. 2006;78(1):89–102. doi: 10.1086/498985 ; 16385452 10.1086/498985 PMC1380226

[21] Kingsley DM, Kozarsky KF, Hobbie L, Krieger M. Reversible defects in O-linked glycosylation and LDL receptor expression in a UDP-Gal/UDP-GalNAc 4-epimerase deficient mutant. Cell. 1986;44(5):749–59. Epub 1986/03/14. . 3948246 10.1016/0092-8674(86)90841-x

[22] Krieger M, Brown MS, Goldstein JL. Isolation of Chinese hamster cell mutants defective in the receptor-mediated endocytosis of low density lipoprotein. Journal of molecular biology. 1981;150(2):167–84. Epub 1981/08/05. . 6275086 10.1016/0022-2836(81)90447-2

[23] Krieger M, Reddy P, Kozarsky K, Kingsley D, Hobbie L, Penman M. Analysis of the synthesis, intracellular sorting, and function of glycoproteins using a mammalian cell mutant with reversible glycosylation defects. Methods in cell biology. 1989;32:57–84. Epub 1989/01/01. . 2691861 10.1016/s0091-679x(08)61167-x

[24] Jang-Lee J, North SJ, Sutton-Smith M, Goldberg D, Panico M, Morris H, et al Glycomic profiling of cells and tissues by mass spectrometry: fingerprinting and sequencing methodologies. Methods in enzymology. 2006;415:59–86. Epub 2006/11/23. doi: 10.1016/S0076-6879(06)15005-3 . 17116468 10.1016/S0076-6879(06)15005-3

[25] Ceroni A, Maass K, Geyer H, Geyer R, Dell A, Haslam SM. GlycoWorkbench: a tool for the computer-assisted annotation of mass spectra of glycans. Journal of proteome research. 2008;7(4):1650–9. Epub 2008/03/04. doi: 10.1021/pr7008252 . 18311910 10.1021/pr7008252

[26] Stansell E, Canis K, Haslam SM, Dell A, Desrosiers RC. Simian immunodeficiency virus from the sooty mangabey and rhesus macaque is modified with O-linked carbohydrate. J Virol. 2011;85(1):582–95. Epub 2010/10/22. doi: 10.1128/JVI.01871-10 ; 20962077 10.1128/JVI.01871-10 PMC3014205

[27] Stansell E, Panico M, Canis K, Pang PC, Bouché L, Binet D, et al Gp120 on HIV-1 Virions Lacks O-Linked Carbohydrate. PLoS One. 2015;10(4). doi: 10.1371/journal.pone.0124784 ; 25915761 10.1371/journal.pone.0124784 PMC4410959

[28] Varki A, Cummings RD, Aebi M, Packer NH, Seeberger PH, Esko JD, et al Symbol Nomenclature for Graphical Representations of Glycans. Glycobiology. 2015;25(12):1323–4. Epub 2015/11/07. doi: 10.1093/glycob/cwv091 ; 26543186 10.1093/glycob/cwv091 PMC4643639

[29] Wohlers TM, Christacos NC, Harreman MT, Fridovich-Keil JL. Identification and characterization of a mutation, in the human UDP-galactose-4-epimerase gene, associated with generalized epimerase-deficiency galactosemia. American journal of human genetics. 1999;64(2):462–70. Epub 1999/02/11. doi: 10.1086/302263 ; 9973283 10.1086/302263 PMC1377755

[30] Timson DJ, Lindert S. Comparison of dynamics of wildtype and V94M human UDP-galactose 4-epimerase-A computational perspective on severe epimerase-deficiency galactosemia. Gene. 2013;526(2):318–24. Epub 2013/06/05. doi: 10.1016/j.gene.2013.05.027 ; 23732289 10.1016/j.gene.2013.05.027 PMC3763920

[31] Schulz JM, Ross KL, Malmstrom K, Krieger M, Fridovich-Keil JL. Mediators of galactose sensitivity in UDP-galactose 4'-epimerase-impaired mammalian cells. The Journal of biological chemistry. 2005;280(14):13493–502. Epub 2005/02/11. doi: 10.1074/jbc.M414045200 . 15701638 10.1074/jbc.M414045200

