# Supplementary figures and images for: HEK293T cell lines defective for O-linked glycosylation
Source: PLoS One. 2017 Jun 27;12(6):e0179949. doi: 10.1371/journal.pone.0179949 (PMC5487050; doi:10.1371/journal.pone.0179949)

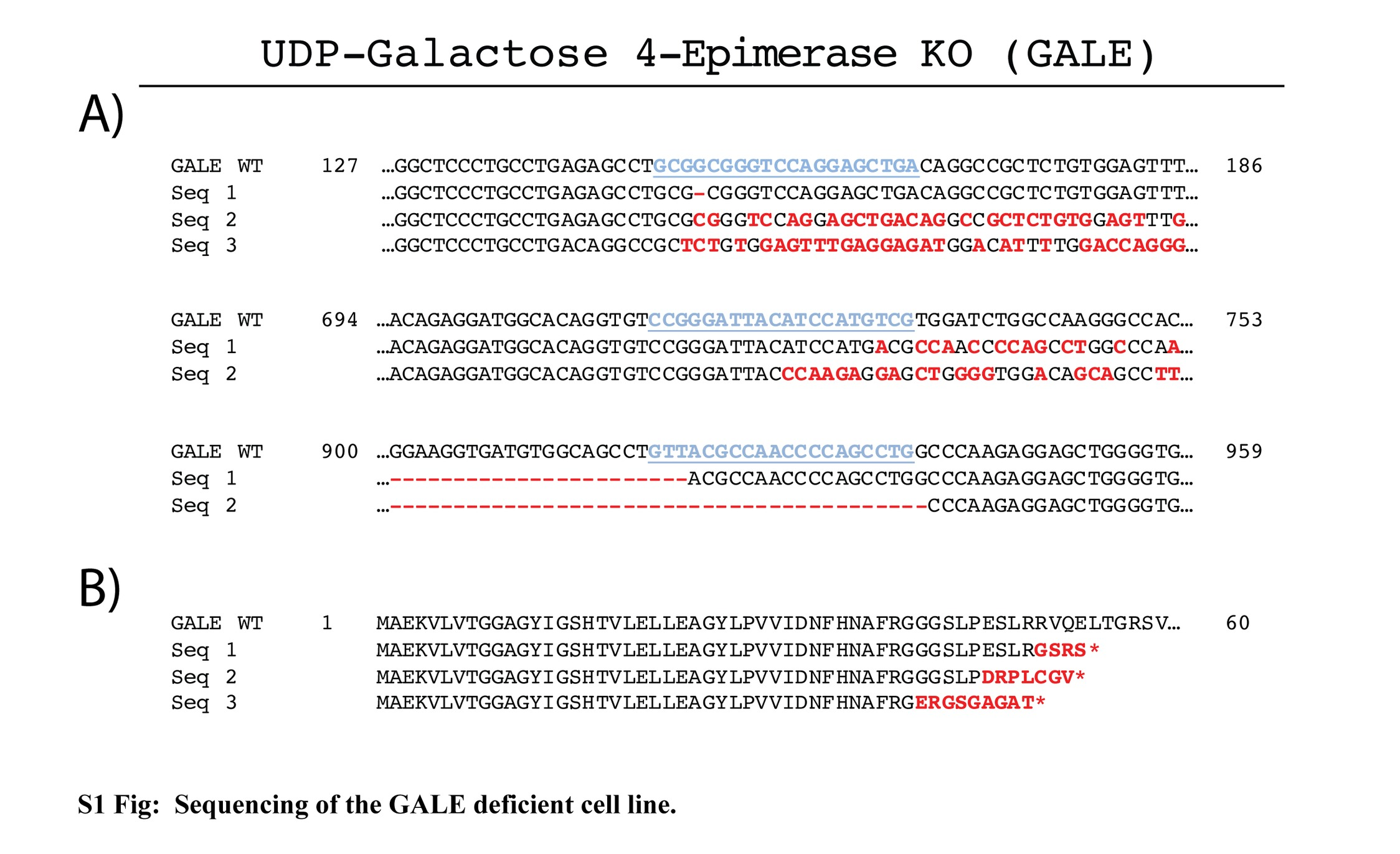

Supplement: S1 Fig — A) Three unique guide RNAs (light blue, underlined nucleotides) were designed to target GALE and generate a deficient HEK293T cell line. To confirm the cell lines, we extracted RNA from 5x10^6^ HEK293T cell lines that had been knocked out for GALE and deemed promising by western blot. cDNA was generated using the superscript IV first-strand synthesis system and amplified a region of the GALE mRNA transcript that is conserved across all transcript isoforms. We performed a PCR cleanup and cloned the GALE transcripts using the Zero Blunt TOPO PCR Cloning Kit. Because HEK293T cells are a tetraploid cell line, we sent 15 unique colonies for Sanger sequencing to ensure that no functional transcripts were present. No functional transcripts were identified. Three unique nucleotide sequences were found in the area of gRNA #1, two were found in the area of gRNA #2, and two were found in the area of gRNA #3. B) Upon conversion of these GALE mRNA transcripts to protein sequences, premature stop codons were consistently identified prior to the 60th amino acid of the GALE protein. (TIF) [file pone.0179949.s001.tif]

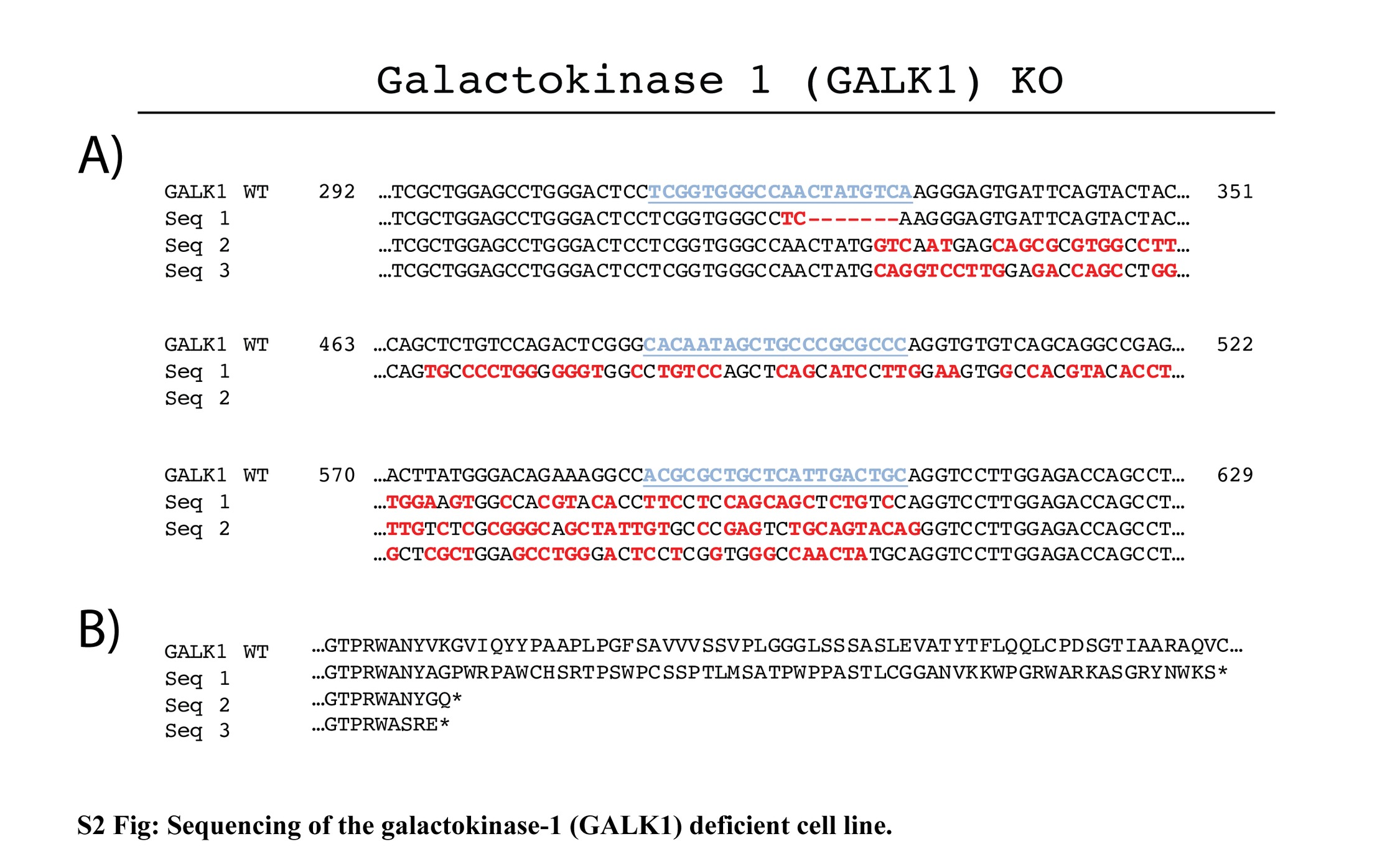

Supplement: S2 Fig — A) Three unique guide RNAs (light blue, underlined nucleotides) were designed to target GALK1 and generate a deficient HEK293T cell line. To confirm the cell lines, we extracted RNA from 5x10^6^ HEK293T cell lines that had been knocked out for GALK1. We created cDNA using the superscript IV first-strand synthesis system and amplified a region of the GALK1 mRNA transcript that is conserved across all transcript isoforms. We performed a PCR cleanup and cloned the GALK1 transcripts using the Zero Blunt TOPO PCR Cloning Kit. Because HEK293T cells are a tetraploid cell line, we sent 15 unique colonies for Sanger sequencing to ensure that no functional transcripts were present in the cell line. Mutations were present in close proximity to all three gRNA sites. We observed three unique mutated transcripts in the region of gRNA #1, one unique transcript in the region of gRNA #2, and three unique transcripts in the region of gRNA #3. No wild-type GALK1 transcripts were found in any of the clones. B) Translation of the mRNA sequences yielded only non-functional, truncated protein sequences. Three unique protein sequences were all identified, each one containing a premature stop codon. (TIF) [file pone.0179949.s002.tif]

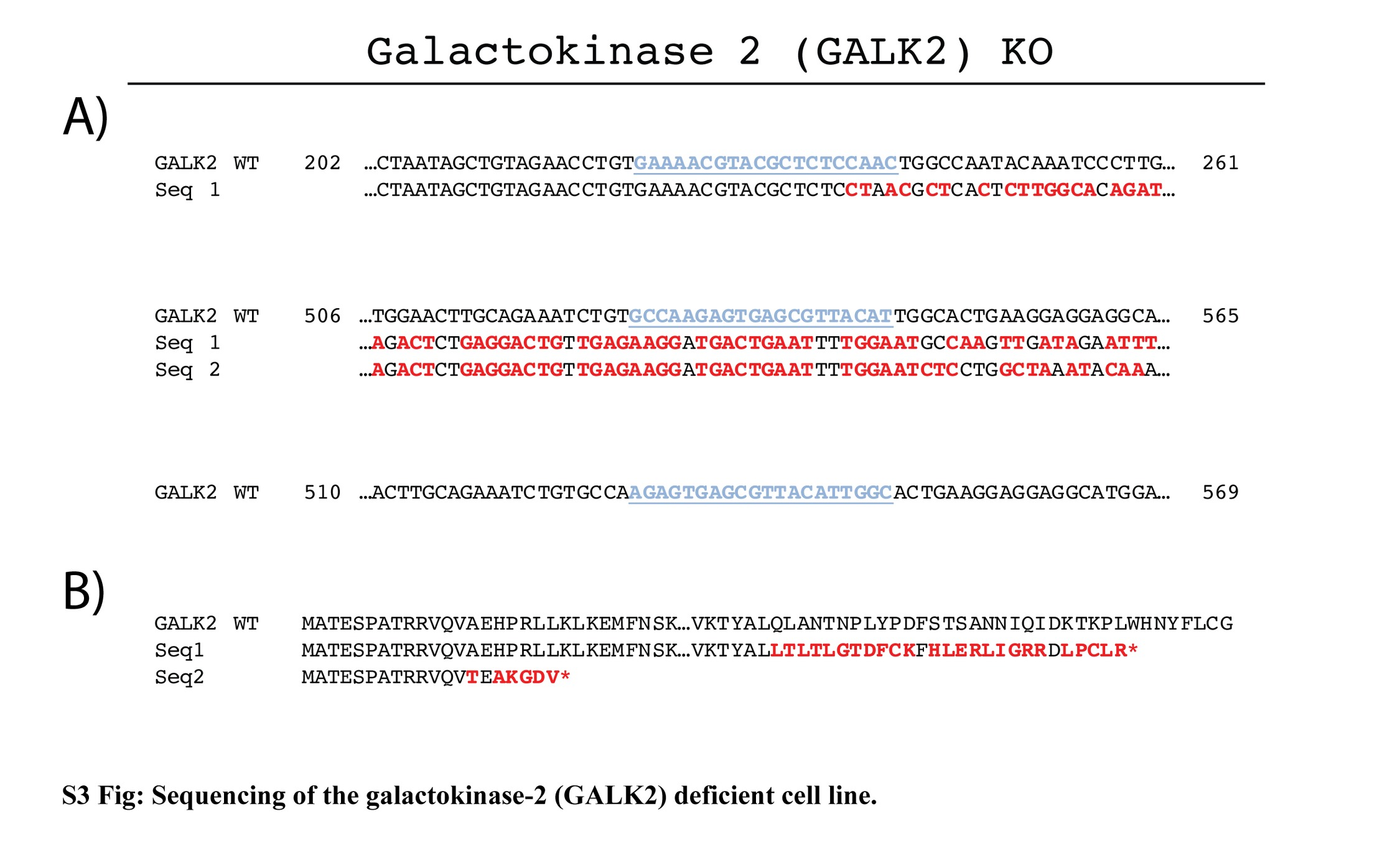

Supplement: S3 Fig — A) Three unique guide RNAs (light blue, underlined nucleotides) were designed to target GALK2 and generate a deficient HEK293T cell line. To confirm the cell lines, we extracted RNA from 5x10^6^ HEK293T cell lines that had been knocked out for GALK2 and deemed promising by western blot. We created cDNA using the superscript IV first-strand synthesis system and amplified a region of the GALK2 mRNA transcript that is conserved across all transcript isoforms. We performed a PCR cleanup and cloned the GALK2 transcripts using the Zero Blunt TOPO PCR Cloning Kit. Because HEK293T cells are a tetraploid cell line, we sent 15 unique colonies for Sanger sequencing to ensure that no functional transcripts were present. All of the clones displayed an identical set of nucleotide mutations in the area of the first gRNA, while two highly similar yet unique sets of mutations were identified circa the second gRNA. Interestingly, no mutations were found near the third gRNA. B) Upon conversion of the mRNA transcripts to protein sequences, premature stop codons leading to truncated proteins were observed across all clones. (TIF) [file pone.0179949.s003.tif]

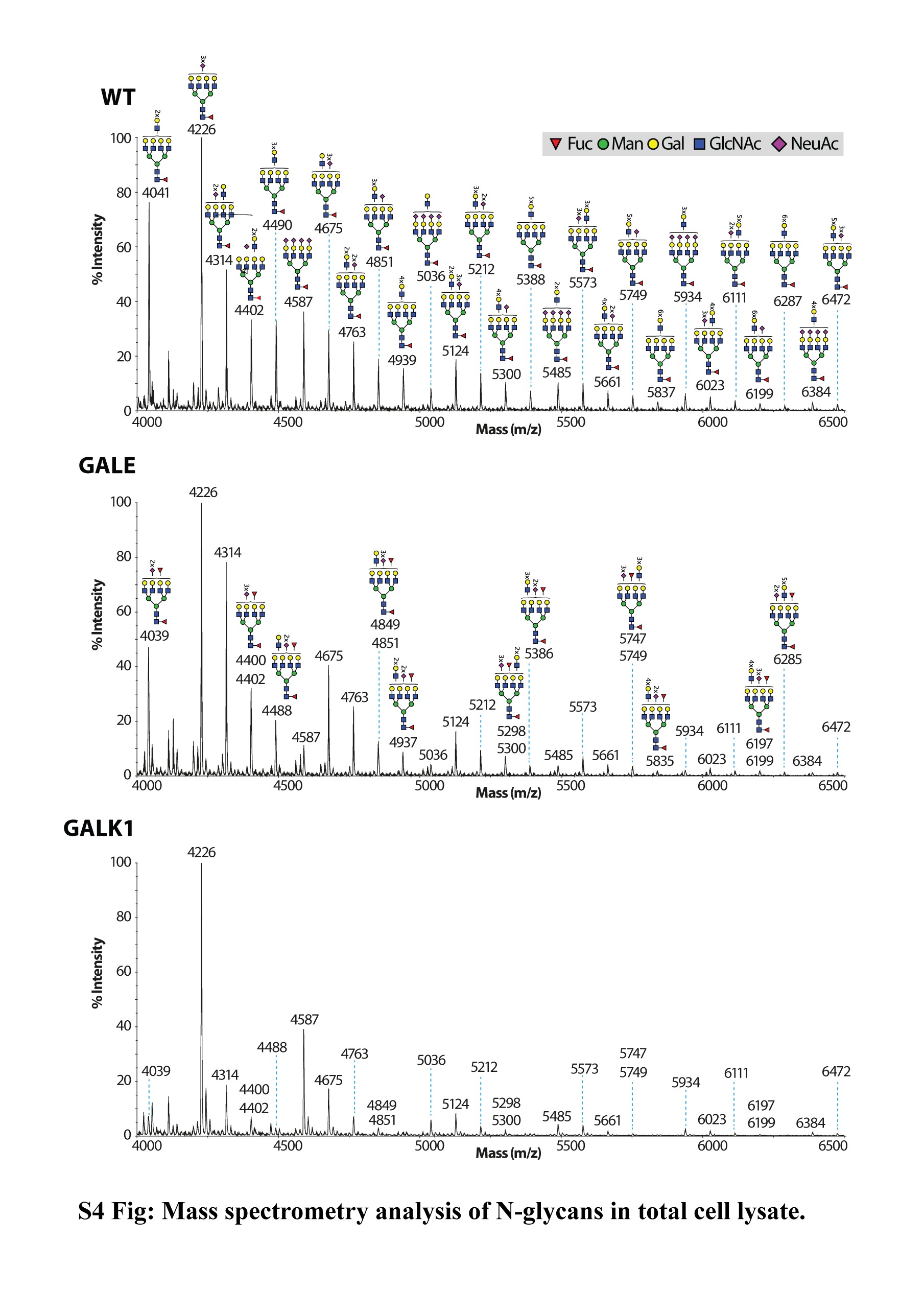

Supplement: S4 Fig — Matrix-assisted laser desorption/ionization-time-of-flight (MALDI-TOF) mass spectra profiles of high molecular weight permethylated N-glycans isolated from total cell lysate of HEK293T (upper panel), GALE KO (middle panel), GALK1 KO (lower panel) cells. All molecular ions are [M+Na]+. Profiles of N-glycans are from the 50% acetonitrile fraction from a C18 sep-pak. The sugar symbols are those as described in (Varki et al., 2015). Putative structural based on monosaccharide composition (obtained by MALDI-TOF MS), fragmentation analyses (MALDI-TOF/TOF MS/MS), and knowledge of glycan biosynthetic pathways. For non-annotated peaks on GALE KO and GALK1 KO see structural assignments on HEK293T. (TIF) [file pone.0179949.s004.tif]
